# Human Skeletal Muscle Mitochondrial Uncoupling Is Associated with Cold Induced Adaptive Thermogenesis

**DOI:** 10.1371/journal.pone.0001777

**Published:** 2008-03-12

**Authors:** Sander L. J. Wijers, Patrick Schrauwen, Wim H. M. Saris, Wouter D. van Marken Lichtenbelt

**Affiliations:** Department of Human Biology, Nutrition and Toxicology Research Institute Maastricht (NUTRIM), Maastricht University, The Netherlands; University of Parma, Italy

## Abstract

**Background:**

Mild cold exposure and overfeeding are known to elevate energy expenditure in mammals, including humans. This process is called adaptive thermogenesis. In small animals, adaptive thermogenesis is mainly caused by mitochondrial uncoupling in brown adipose tissue and regulated via the sympathetic nervous system. In humans, skeletal muscle is a candidate tissue, known to account for a large part of the epinephrine-induced increase in energy expenditure. However, mitochondrial uncoupling in skeletal muscle has not extensively been studied in relation to adaptive thermogenesis in humans. Therefore we hypothesized that cold-induced adaptive thermogenesis in humans is accompanied by an increase in mitochondrial uncoupling in skeletal muscle.

**Methodology/Principal Findings:**

The metabolic response to mild cold exposure in 11 lean, male subjects was measured in a respiration chamber at baseline and mild cold exposure. Skeletal muscle mitochondrial uncoupling (state 4) was measured in muscle biopsies taken at the end of the respiration chamber stays. Mild cold exposure caused a significant increase in 24h energy expenditure of 2.8% (0.32 MJ/day, range of −0.21 to 1.66 MJ/day, p<0.05). The individual increases in energy expenditure correlated to state 4 respiration (p<0.02, R^2^ = 0.50).

**Conclusions/Significance:**

This study for the first time shows that in humans, skeletal muscle has the intrinsic capacity for cold induced adaptive thermogenesis via mitochondrial uncoupling under physiological conditions. This opens possibilities for mitochondrial uncoupling as an alternative therapeutic target in the treatment of obesity.

## Introduction

Obesity is reaching epidemic forms, and is expected to have a major impact on public health in the Western world [1]–[3]. It develops when energy intake exceeds energy expenditure for a prolonged period of time. Despite the strategies aimed at reducing energy intake, the prevalence of obesity is still rising. Therefore, augmenting energy expenditure is an attractive alternative strategy to tackle the obesity epidemic. Mild cold exposure is known to elevate energy expenditure in mammals, including humans. This regulated increase in energy expenditure is called adaptive thermogenesis [4].

In small mammals brown adipose tissue is responsible for the major part of adaptive thermogenesis via the action of the brown-adipose tissue specific uncoupling protein 1 (UCP-1), controlled by the sympathetic nervous system [5]. This protein is able to lower the proton gradient across the mitochondrial inner membrane and bypasses ATP-synthase (i.e. mitochondrial uncoupling), thereby preventing ATP production and dissipating energy as heat [5].

Although the impact of brown adipose tissue in adult humans is questionable and still under debate [6], [7], evidence can be obtained for the existence of adaptive thermogenesis. For example, it has been shown that a fixed excess of calories by overeating causes a three-fold range in weight gain among subjects [8]. Also after mild cold exposure a large range in the increase in energy expenditure has been found [9]. In humans, it is shown that skeletal muscle can be responsible for a 40% increase in total body energy expenditure after epinephrine infusion [10]. However, no data on human skeletal muscle mitochondrial uncoupling in relation to adaptive thermogenesis are available as yet.

Here we hypothesize that skeletal muscle has the intrinsic capacity for adaptive thermogenesis in humans via mitochondrial uncoupling. We investigated energy expenditure, mitochondrial uncoupling, and expression of selected proteins after cold exposure in eleven healthy male subjects.

## Results

Eleven lean male subjects stayed twice in a respiration chamber: once for a baseline measurement (energy balance, 22°C) and once during mild cold exposure (energy balance, 16°C). After each stay a muscle biopsy was taken.

**Figure 1:**
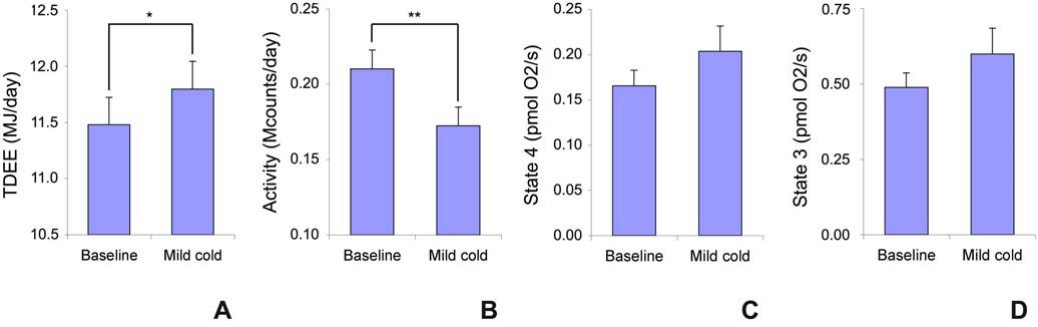
Baseline and mild cold values of a) total daily energy expenditure, b) activity, c) state 4 respiration, and d) state 3 respiration. The figures show mean (±SEM) in both situations. * p<0.05, ** p<0.01

Total daily energy expenditure (TDEE) was measured for the last 24 hours of each stay. TDEE increased significantly with 2.8% (0.32 MJ/day, z = 1.96, p<0.05) (figure 1a). The inter-subject variability in the change in TDEE was large (range of −0.21 to 1.66 MJ/day). The increase in TDEE was not explained by increased physical activity: in fact mild cold exposure resulted in a significant decrease in physical activity (−39 kcounts/day, z = −2.67, p<0.01) (figure 1b).

**Figure 2:**
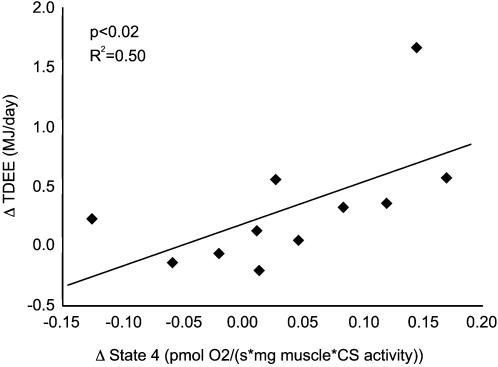
Regression of increases in total daily energy expenditure (MJ/day) and state 4 respiration (pmol O_2_/(s·mg muscle·CS activity)) (p<0.02, R^2^ = 0.50).

Human skeletal muscle mitochondrial uncoupling was assessed using high-resolution respirometry (see methods). Oxygen consumption was measured in permeabilized muscle fibers. Addition of mitochondrial substrates and ADP leads to a basal level of respiration, called state 3. After addition of oligomycin, ATP-synthase is blocked and the remaining oxygen consumption can only be due to proton leakage. This remaining respiration is called state 4, and is a measure of mitochondrial uncoupling. Mitochondrial respiration measurements have been corrected for citrate synthase activity, as a measure of mitochondrial content. Citrate synthase activity did not differ on average between baseline and cold exposure measurements. On average, neither state 4 nor state 3 did increase after mild cold exposure (respectively z = −1.38, p = 0.168 and z = −1.38, p = 0.168) (figure 1c and 1d). However, interindividual differences were large (state 4: range of −0.12 to 0.17 pmol O_2_/(s·mg muscle·CS activity); state 3: range of −0.22 to 0.62 pmol O_2_/(s·mg muscle·CS activity)). Changes in TDEE correlated significantly to state 4 respiration (p = 0.015, R^2^ = 0.50) (figure 2). No correlation of TDEE with state 3 was observed.

Since UCP-3 content and mitochondrial density can change muscular energy expenditure, UCP-3 and oxidative phosphorylation protein content and citrate synthase activity have been measured in a separate set of 9 subjects under identical experimental conditions. UCP-3 and oxidative phosphorylation protein complex I, II, III, IV, and V expression did not change significantly (table 1).

**Table 1:** protein expression data

	Baseline	Cold	Cold- Baseline		
	Mean±SEM		Delta	Z-score	P
UCP 3 (AU)	22.24 ±2.55	30.21±4.95	7.97	−0.91	0.36
Complex I (AU)	1.32±0.24	2.10±0.69	0.78	−0.17	0.17
Complex II (AU)	14.07±1.51	17.07±2.65	3.00	−0.84	0.40
Complex III (AU)	17.82±2.32	20.91±3.73	3.09	−0.28	0.78
Complex IV (AU)	9.37±1.04	10.58±1.62	1.21	−0.14	0.89
Complex V (AU)	33.12±2.82	32.63±2.53	−0.49	−0.28	0.78

AU, arbitrary units

## Discussion

The results show that human adaptive thermogenesis in response to cold exposure is accompanied by and related to mitochondrial uncoupling in skeletal muscle. Fifty percent of the variation in the individual increases in energy expenditure upon cold exposure can be explained by mitochondrial uncoupling

Since mitochondrial protein expression and citrate synthase activity did not increase after mild cold exposure, an augmentation of mitochondrial density can be ruled out [11]. Also an increase in physical activity can be excluded, since radar counts were lower–and not higher–after cold exposure. Shivering can be excluded too; subjects reported they did not perceive any shivering at all. Furthermore, in a previous study in the same study setting, this has been validated by electromyogram measurements [12].

The process of skeletal muscle mitochondria uncoupling is thus likely to contribute to the increase in energy expenditure. Previously, it has been shown that mitochondrial uncoupling in humans could be stimulated. Infusion of T3, which is a known factor involved in cellular respiration, resulted in higher mitochondrial uncoupling [13]. However, this study for the first time shows that environmental factors affect both adaptive thermogenesis and mitochondrial uncoupling.

The increase in energy expenditure, 0.32 MJ/day (−0.20 to 1.66 MJ/day), is comparable to data from literature [9], [14], [15]. This increase is considerably smaller than the increase in energy expenditure observed in small mammals after cold exposure (two- to fourfold) [16]. However, a small difference in energy expenditure for a prolonged period might contribute largely to weight gain. A diminished adaptive thermogenesis has indeed been identified as a risk factor for obesity [17]. Therefore, our results indicate that mitochondrial uncoupling in human skeletal muscle may be a target for therapies to prevent obesity.

In the past, drugs have been developed to treat obesity, for example 2,4-Dinitrophenol, without insight in the mechanisms behind. This drug, which has a major effect on the uncoupling of oxidative phosphorylation, turned out to be lethal due to hyperthermia and has been banned in 1938, but is still used in illegal circuits due to its marked effects on body weight [18]. The present study shows that skeletal muscle has the intrinsic capacity of regulated mitochondrial uncoupling. Research should aim to target this intrinsic uncoupling process in the mitochondrial membrane without disrupting the complete membrane as is the case with the use of 2,4-Dinitrophenol to increase thermogenesis and thus weight loss.

To gain more insight in the processes underlying cold-induced mitochondrial uncoupling, further research is needed to unravel the molecular nature of these processes in human skeletal muscle. Since UCP-1 and also the more recently discovered uncoupling proteins UCP-2, UCP-4, and UCP-5 are not present in human muscle [19]–[21], other candidate actors have to be found. UCP-3 is present in human muscle, but it is currently believed to play a role in fatty acid metabolism rather than in direct mitochondrial uncoupling [22]. This is underlined by our finding that UCP-3 protein expression was not upregulated during cold exposure. Therefore, so far unknown proteins may be found that are able to provoke mitochondrial uncoupling. Proteomics and genomics approaches would be methods of choice, since with these methods no prior targets have to be determined.

Acclimatization to cold is known to have large effects on adaptive thermogenesis. Subjects regularly exposed to mild cold have a higher adaptive thermogenesis capacity [12]. This could be of benefit in situations of overfeeding, since cold-induced adaptive thermogenesis is related to overfeeding induced adaptive thermogenesis, as we have shown recently [23]. Therefore, the results indicate that regular cold exposure might be beneficial in body weight regulation via an increase in the skeletal muscle uncoupling capacity.

In conclusion, human skeletal muscle has the intrinsic capacity for mitochondrial uncoupling. Cold-induced increases in energy expenditure are accompanied by an increase in skeletal muscle mitochondrial uncoupling.

## Materials and Methods

Eleven lean male subjects (height: 184±3 cm; body mass: 77.7±2.7 kg; BMI: 22.9±0.6 kg/m^2^; age: 21.7±0.6 years), not using any medications and not seen by a MD in the last three years participated in this study. Subjects stayed twice in the respiration chambers of the department of Human Biology, Maastricht University, wearing standardized clothing. The two conditions are defined as follows: 1) baseline: 34 hours measurement of energy expenditure at energy balance, comfortable temperature (22°C); 2) cold exposure for 82 hours in energy balance, mild cold without shivering (16°C). Macro-nutrient composition was equal in both conditions: 47, 38, and 15% energy from carbohydrate, fat, and protein, respectively. After each respiration chamber stay a muscle biopsy was taken, from M. vastus lateralis according to the technique of Bergström [24]. A part of the biopsy was stored in a preservation medium for mitochondrial uncoupling measurement, the rest of the biopsy was quickly frozen in liquid nitrogen for protein quantification and CS activity analyses.

The Medical Ethical Committee of Maastricht University/University Hospital Maastricht approved the protocol. All participants provided oral and written informed consent.

### Energy Expenditure

The respiration chambers are whole body calorimeters annex climate chambers [25]. Total daily energy expenditure (TDEE) was determined from the subjects' O_2_ consumption, CO_2_ production and urine nitrogen excretion according to the Weir equation [26] during the last 24 hours of the respiration chamber stay. Physical activity was monitored during the last day of each stay using a radar system based on the Doppler principle, as described previously [27].

### Mitochondrial uncoupling

Mitochondrial respirometry measurements have been made in permeabilized muscle fibers. We have chosen for permeabilized fibers instead of isolated mitochondria since the mitochondria then remain in a more physiological environment.

Muscle tissue was prepared for mitochondrial respiration measurements as follows: after taking the muscle biopsy, the sample was stored in an ice-cold preservation buffer (10 mM Ca-EGTA buffer, 0.1 µM free calcium, 20 mM imidazole, 20 mM taurine, 50 mM K-MES, 0.5 mM DTT, 6.56 mM MgCl_2_, 5.77 mM ATP, 15 mM phosphocreatine, pH 7.1). Individual fibre bundles were separated using sharp needles to increase surface area. Subsequently fibers were permeabilized according to the method designed by Veksler et al [28] using saponin. After that, muscle fibers were washed in a respiration medium (0.5 mM EGTA, 3 mM MgCl_2_, 60 mM K-lactobionate, 20 mM Taurine, 10 mM KH_2_PO_4_, 20 mM HEPES, 110 mM Sucrose and 1 g/l BSA, pH 7.1).

**Figure 3:**
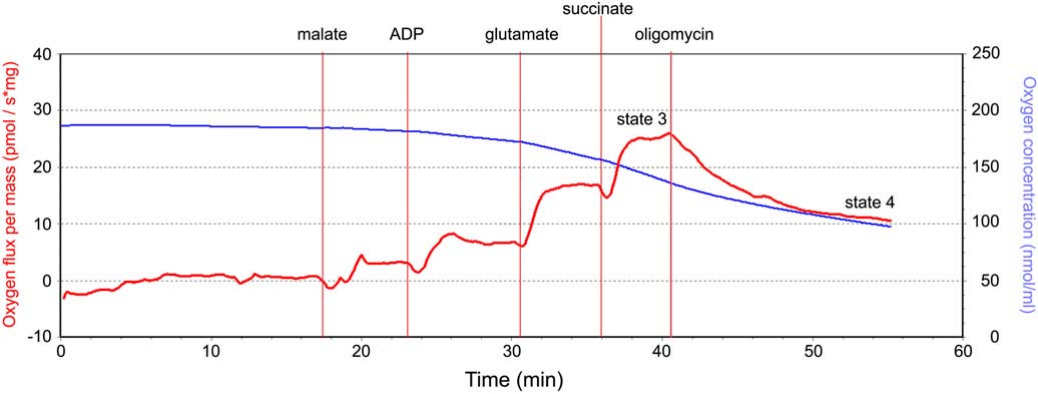
A sample trace of the Oxygraph-2K. The blue line indicates the oxygen concentration in the sample. The red line indicates its derivative, the oxygen consumption by the sample in the respiration medium. The responses to the addition of malate (5 mM), ADP (2 mM), glutamate (10 mM), succinate (10 mM), and oligomycin (1 µg/ml) can be seen. After succinate addition, state 3 was achieved. After oligomycin addition, state 4 was achieved.

Mitochondrial respiration measurements were carried out with 2–6 mg of permeabilized muscle fibre in the same respiration buffer in the Oxygraph-2K (Oroboros, Austria). Subsequently, malate (5 mM), ADP (2 mM), glutamate (10 mM), succinate (10 mM), and oligomycin (1 µg/ml) were added (figure 3). After succinate addition state 3 was achieved: the maximal oxygen consumption with production of ATP. After oligomycin addition, ATP-synthase was blocked and state 4 was measured: the oxygen consumption without ATP-production. Measurements have been normalized for the wet weight of added muscle tissue. Furthermore, respiration measurements have been corrected for citrate synthase activity (see below) to correct for mitochondrial content.

### Protein quantification and CS activity

For UCP3 protein determination, muscle biopsies were homogenized in ice-cold Tris-EDTA buffer at pH 7.4, and then the homogenates were sonicated for 15 seconds. Subsequently, two volumes of each skeletal muscle homogenate and one volume of SDS-sample buffer were boiled for 4 min. Next, 13% polyacrylamide gels containing 0.1% SDS were loaded with equal amounts of protein from each sample, and electrophoresis was performed using a Mini-Protean 3 Electrophoresis Cell (Bio-Rad Laboratories, Hercules, CA). After gel electrophoreses, the gel was scanned, and the optical density of the 43-kDa band, previously immuno-identified to represent actin, was assessed. Then, a second gel was prepared and loaded with the sample volume (which had been recalculated based on the optical density of the actin band), after which Western blotting was performed using a Mini Trans-Blot Electrophoretic Transfer Cell (Bio-Rad Laboratories) as described previously [29]. We used a rabbit polyclonal UCP3 antibody (code 1331; kindly provided by LJ Slieker, Eli Lilly) prepared against a 20–amino acid (aa) peptide (human sequence aa 147–166). For a detailed description of the selectivity and specificity checks see previous reports [29], [30]. Cytochrome C level, as a marker of mitochondrial content, was measured comparably using a rabbit polyclonal cytochrome c antibody (BD PharMingen, Woerden, NL). The ND6 subunit of complex I, the 30 kDa Ip subunit of complex II, the 47 kDa core protein 2 of complex III, subunit II of cytochrome C oxidase (complex IV) and the alpha subunit of the F1F0 ATP synthase (complex V) were measured using monoclonal antibodies (MitoSciences, Oregon, USA). All proteins were expressed as arbitrary units (AU). Citrate synthase activity has been measured following the method of Shephard and Garland [31].

### Statistical analyses

Statistical analyses were done using SPSS 11 for Mac OS X. Data were reported as mean±SEM. Comparisons between groups were made using the non parametric Wilcoxon signed rank test. Correlation tests have been performed using Spearman correlation. A p-value smaller than 0.05 was considered to be statistically significant.
